# *Plasmodium* translationally repressed gene products are essential for parasite development and malaria transmission

**DOI:** 10.1186/1475-2875-11-S1-P85

**Published:** 2012-10-15

**Authors:** Jorge M Santos, Pavitra N Rao, Ana Guerreiro, Leonor Pinho, Céline K Carret, Blandine MD Franke-Fayard, Thomas J Templeton, Chris J Janse, Gunnar R Mair

**Affiliations:** 1 Unidade de Parasitologia Molecular, Instituto de Medicina Molecular, Av. Prof. Egas Moniz, 1649-028 Lisbon, Portugal; 2 Department of Microbiology and Immunology, Weill Cornell Medical College, New York 10021, USA; 3 Leiden Malaria Research Group, Department of Parasitology, Leiden University Medical Center, 2333 ZA Leiden, Netherlands

## Background

The sexual and ookinete development of *Plasmodium* relies on the translation of mRNAs supplied maternally in the macro-gametocyte as translationally repressed transcripts [1]. Translational repression depends on the interaction of mRNAs and RNA binding proteins such as DOZI and CITH; their absence results in mRNA destabilisation and developmental arrest of the parasite in the mosquito midgut [2]. Among the repressed mRNAs ~40 encode for potential surface molecules or adhesins. While some are well-characterised (e.g. P25 and P28), most are putative with no known function or homology. *pb25/pb28* gene disruption severely impairs parasite development [3] while the presence of anti-P25 and anti-P28 antibodies in a blood meal reduces mosquito infection [4,5]. A P25-based transmission blocking vaccine (TBV) has reached human phase 1 clinical trials but results have not been fully satisfactory [6]. For this reason, novel antigens are being pursued as targets of malaria TBVs.

## Materials and methods

RIP-Chip and RT-PCR of immunoprecipitated DOZI and CITH mRNPs from the rodent malaria parasite *P.berghei* were used to identify molecules with clear surface targeting signals that are associated with P body-like mRNPs. Targeted gene deletion and GFP-tagging was used to identify the function, sub-cellular localisation and expression patterns of these proteins. To study the development of knock-out parasite lines, *Anopheles stephensi* mosquitoes were allowed to feed on infected mice and infection was quantified at the oocyst and sporozoite stages. *In silico* analysis identified highly immunogenic peptides in each protein; they were concatenated as codon-optimised, chimerical His_6_-tagged fusion proteins for heterologous expression to be used in transmission blocking assays.

## Results

22 mRNAs encoding for surface proteins were shown to be associated with both DOZI- and CITH-defined mRNPs. These include *pb25*, *pb28*, the entire *pb-fam-5* family, as well as 12 uncharacterised gene products with orthologs in *P. falciparum* and *P. vivax;* the latter were targeted for gene deletion. Several of the knock-out mutants show a clear impairment in mosquito stage development, either at the oocyst or sporozoite levels. Some of these completely fail to transmit to naïve mice. Full length and concatenated versions (superantigens) of these proteins were expressed in *Escherichia coli* BL21. Superantigen expression was achieved more easily and in higher yields than full-length proteins.

## Conclusions

Here we identify novel *P. berghei* translationally repressed mRNAs that encode for mosquito stage surface proteins and are important for parasite development within its vector. Parasites lacking some of these proteins fail to transmit to naïve hosts, and are therefore attractive targets for novel transmission blocking vaccines. Heterologous expression of *Plasmodium* protein is frequently a challenging task due to their disordered nature and the A/T richness of the genome. We used codon optimisation to compensate for A/T rich genes and developed a superantigen strategy to combine the most immunogenic regions of several proteins while avoiding their hydrophobic domains. The results show that our superantigens are more easily expressed in bacteria, and in higher amounts. This will enable us to use them in transmission blocking assays, targeting more than one parasite surface protein at the same time.
